# Calcium Fluoride Precipitation and Deposition From 12 mmol/L Fluoride Solutions With Different Calcium Addition Rates

**DOI:** 10.6028/jres.114.021

**Published:** 2009-10-01

**Authors:** M Markovic, S Takagi, LC Chow, S Frukhtbeyn

**Affiliations:** American Dental Association Foundation, Paffenbarger Research Center, National Institute of Standards and Technology, Gaithersburg, MD 20899

**Keywords:** calcium addition rate, calcium fluoride, fluoride deposition, fluoride solution, precipitation

## Abstract

The effects of different Ca-addition rates on calcium fluoride (CaF_2_) precipitation and deposition were investigated in 12 mmol/L sodium fluoride solutions to which 0.1 mol/L calcium chloride solution was continuously added at average rates of (5, 7.5, 10, 12.5, 15 or 20) mmol L^−1^ min^−1^. The changes in ionic fluoride and calcium concentrations, as well as turbidity, were continuously recorded by F and Ca electrodes, and a fiber optic based spectrophotometer, respectively. The F^−^ concentration decreased and turbidity increased with time indicating precipitation of CaF_2_. For the systems with Ca-addition rates of (5, 7.5, 10, 12.5, 15, and 20) mmol L^−1^ min^−1^, the 1 min CaF_2_ depositions in the model substrate (cellulose filter paper, pores 0.2 µm) expressed as mean ± SD of deposited F per substrate surface area were (3.78 ± 0.31, 11.45 ± 0.89, 9.31 ± 0.68, 8.20 ± 0.56, 6.63 ± 0.43, and 2.09 ± 0.28) µg/cm^2^, respectively (*n* = 10 for each group). The 1-min F depositions did not show positive correlation to Ca-addition rates. The lowest 1-min F deposition was obtained in the systems with the highest Ca-addition rate of 20 mmol L^−1^ min^−1^ for which CaF_2_ precipitation rate reached the maximum value of 0.31 mmol L^−1^ s^−1^ almost immediately after beginning of reaction (6 s). The largest 1-min F depositions were obtained from the systems with Ca addition rates of (7.5 to 12.5) mmol L^−1^ min^−1^ in which CaF_2_ precipitation rates continuously increased reaching the maximum values of (0.13 to 0.20) mmol L^−1^ s^−1^ after (18 to 29) s, respectively. The 1-min F depositions were greatly enhanced in comparison with the control F solutions that did not have continuous Ca-addition. This indicates that continuous Ca addition that controls the rate of CaF_2_ formation could be a critical factor for larger F depositions from F solutions. The efficacy of conventional F mouthrinses could be improved with addition of a substance that continuously releases Ca.

## 1. Introduction

The bioavailable fluoride (F) deposits in the oral environment are primarily in the form of calcium fluoride (CaF_2_) or CaF_2_-like precipitates [1–5]. These CaF_2_ deposits in the dental plaque become an oral F reservoir [6–8], which gradually releases F ions into saliva [9] that tends to maintain a constant elevated level of F. Persistent elevated F in oral fluids has been shown to have a profound effect in caries protection [10–12].

The CaF_2_ particles are the major reaction products after a topical fluoride application to tooth surfaces [1–3]. The source of calcium (Ca) necessary for CaF_2_ precipitation after topical F application could be enamel, saliva, plaque fluid, oral mucosa or calculus [13]. It was suggested that CaF_2_ was formed not only on enamel surfaces but also to some extent in the subsurface areas [14,15].

The CaF_2_ particles were also formed during application of two-component rinses that contain fluoride (Na_2_SiF_6_) and calcium (CaCl_2_) sources [16]. Na_2_SiF_6_ instead of NaF was used as the F source because the hydrolysis of SiF_6_^2−^ produces a continuous release of F^−^ into the solution. The Na_2_SiF_6_ type of two-component rinses, containing either a conventional (12 mmol/L F) or reduced (6 mmol/L F) level of F, have been shown to be much more effective than a one-component NaF-based conventional rinse containing 12 mmol/L F in: (a) *in vitro* lesion remineralization [17,18], (b) increasing oral bioavailable F [5,19–21], and most importantly (c) *in situ* lesion remineralization [22,23]. The greater effectiveness of these two-component F-release rinses is due to two factors. Firstly, the two-component rinses provide a high Ca concentration that induces a large amount of CaF_2_ deposits, whereas with a conventional one-component 12 mmol/L NaF rinse only small amounts of CaF_2_ deposits are formed due to the limited amount of oral Ca available [24]. Secondly, the continuous release of F^−^ from SiF_6_^2−^ controls the rate of formation and deposition of CaF_2_ and is the key to high CaF_2_ deposition during the rinse application [16].

Our current goal is to develop a new type of two-component system with fixed NaF concentration and continuous Ca release to produce an enhanced bioavailable CaF_2_ deposition. This novel two-component system with continuous Ca release generated through the dissolution of a suitable Ca salt could have several advantages. Firstly, since it contains NaF (instead of Na_2_SiF_6_) and appropriate Ca salts, which are generally recognized as safe (GRAS) chemicals, it faces fewer regulatory hurdles. Secondly, because the Ca-release rate can be controlled and adjusted by choosing the type, particle size range, and amount of the dissolving Ca salt, a large range of release rates is available, which is needed to counteract the effects of mouthrinse ingredients on CaF_2_ precipitation. The initial step in development of this type of mouthrinse or dentifrice is determination of the Ca-release rate that produces an enhanced CaF_2_ deposition.

In the present study, the CaF_2_ precipitation and deposition were examined in the simple model system with 12 mmol/L NaF solution to which CaCl_2_ solution was continuously added at average rates of (5, 7.5, 10, 12.5, 15 or 20) mmol L^−1^ min^−1^ mimicking dissolution rates of moderately soluble Ca salts. These studies are particularly important for determination of conditions at which the most efficient deposition of CaF_2_ in/on porous model substrate could occur during short-term (1 min) application of Ca-release rinse in which Ca could be continuously released into a NaF solution by dissolution of a solid Ca salt.

## 2. Materials and Methods^1^

### 2.1 Systems With Continuous Calcium Addition

Standard solutions with concentration of 0.1000 mol/L ± 0.0005 mol/L of NaF and CaCl_2_ (Orion Research, Beverly, MA, USA) were used to prepare reactant solutions. The volume of 150 mL of 12 mmol/L NaF solution, with 30 mmol/L KCl as a background electrolyte was placed in a jacketed, 250 mL capacity glass vessel exposed to air and connected to a circulating bath set at 37 ºC. A vigorous stirring at the rate of about 1880 rad/min (300 rotations per minute) was employed using a magnetic stirrer. The 0.1 mol/L CaCl_2_ titrant solution containing 30 mmol/L KCl as background electrolyte was continuously added at a constant rate (in mL/min) with an automatic burette (Dosimat 665, Brinkman Instruments, Westbury, NY, USA). Concomitantly, a 24 mmol/L NaF titrant solution containing 30 mmol/L KCl was added at the same titration rate as the CaCl_2_ titrant solution to compensate for decrease in total F concentration due to continuous increase of reactant solution volume. The titration rate of these two solutions was adjusted to produce the desired average rate of total Ca increase of (5, 7.5, 10, 12.5, 15, or 20) mmol L^−1^ min^−1^ to the reactant solution.

**Fig. 1:**
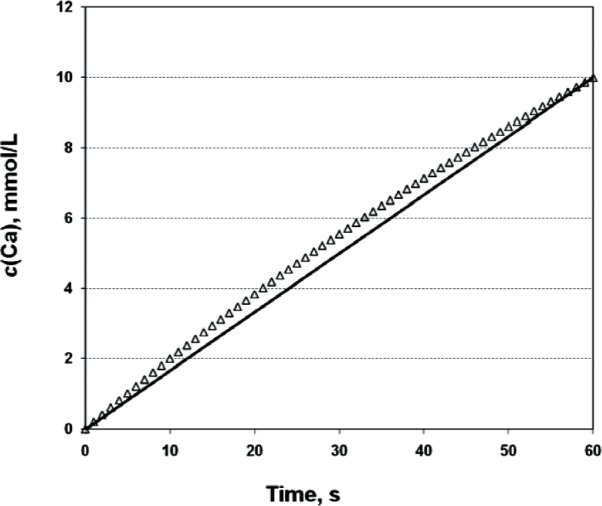
Calculated actual total Ca concentrations in the system with average Ca-addition rate of 10 mmol L^–1^ min^–1^ (Δ), and total calcium concentrations in the hypothetical system with the constant Ca addition rate of 10 mmol L^–1^ min^–1^ (solid line). The difference in Ca concentrations is due to an increase in solution volume because of titrant additions.

The desired average rate of total Ca increase expressed in mmol L^−1^ min^−1^ is defined as the amount of Ca (in mmol) that was added in the total volume of reactant solution (in L) after 1-min titration. The added titrants cause the continuous increase in the total volume of suspension, and therefore the real rate of total Ca increase is not constant and slightly differs from the average rate of total Ca increase rate during the 1-min time interval. As a result, the actual total Ca concentration in the reactant solution does not increase linearly with time (Fig. 1, data points) and is slightly higher than the hypothetical total Ca concentration that increases with a constant rate (Fig. 1, solid line).

A pencil-size combination pH electrode (Model 91-05, Orion Research) and ion selective F-electrode (Model 94-09, Orion Research) and Ca electrode (Model 93-20, Orion Research), were used for determination of pH, and activities (*a*) of F and Ca ions in reactant solutions, respectively. Throughout the experiment, a desktop PC equipped with an analog-to-digital converter card (Model BNC-2080, National Instruments, Austin, TX, USA) recorded one reading per second of the mV signals from the electrodes. The standard F^−^ solutions used for F-electrode calibration contained 0.1 mmol/L to 12 mmol/L NaF and 30 mmol/L KCl as background electrolyte. The standard Ca^2+^ solutions used for Ca-electrode calibration contained 0.1 mmol/L to 20 mmol/L CaCl_2_ and 30 mmol/L KCl. Ionic strengths (*I*) of standard solutions were calculated as *I* = 0.5 Σ*c*_i_*z*_i_^2^ where *c*_i_ and *z*_i_ are ionic concentrations and corresponding relative charges of ions in solution. The activity coefficients (*y*) of F^−^ or Ca^2+^ in each standard solution were calculated using Davies’ Modification [25] of the Extended Debye-Hückel Equation: log *y* = − 0.524*z*_i_^2^[*I*^1/2^/(1 + *I*^1/2^) − 0.3*I*]. The *a*(F^−^) or *a*(Ca^2+^) in standard solutions were calculated from the equation *a* = *yc*. The electrode potentials (*E*) in F^−^ and Ca^2+^ standard solutions (measured in mV) had linear dependence to the logarithmic values of F^−^ and Ca^2+^ activities, respectively. The measured activities of F^−^ and Ca^2+^ in reactant solutions were converted into concentrations and expressed as ionic concentrations *c*(F^−^) and *c*(Ca^2+^) with time, respectively. Based on observed standard deviations for four runs, the standard uncertainty of measured *c*(F^−^) and *c*(Ca^2+^) were ± 3 % and ± 5 %, respectively, of the determined values.

The concentrations of precipitated CaF_2_ amounts in reactant solutions (expressed in mmol/L) at different times were calculated using equation *c*(CaF_2_) = [*c*(F)_total_ − *c*(F^−^) − *c*(CaF^+^)]/2, where *c*(F) total = 12 mmol/L and concentrations of soluble ions (F^−^ and CaF^+^) were calculated with Chemist^©^. The degrees of saturation (*S*) at different times were also calculated with Chemist^©^ using equation *S* = (*IAP*/*K*_sp_)^1/3^, where *IAP* is ionic activity product, *IAP* = *a*(Ca^2+^) · *a*^2^(F^−^), and *K*_sp_ is solubility product of CaF_2_(*pK*_sp_ = 10.41).

The H^+^ or OH^−^ ions were not consumed or produced during CaF_2_ precipitation. Therefore, it was not necessary to have a pH buffer in the solutions.

Turbidity of the suspension was measured as absorbance by a fiber optic spectrophotometer containing a photodetector amplifier (PDA1, World Precision Instruments (WPI), Sarasota, FL, USA), a visible light detector (VISD, WPI), and a miniature dipping probe (DIP-UV, WPI). Standard uncertainty of turbidity was assumed to be ± 0.005 absorbance unit based on observed standard deviations from four runs.

### 2.2 Fluoride Deposition in Model Substrate

Hydrophilic cellulose-ester filter discs with pore sizes of up to 0.2 µm and diameter of 8.0 mm (Bioanalytical Systems, West Lafayette, IN, USA) were used as the porous model substrates [26]. Filter discs (five in each run) were pre-soaked for 1 min in 30 mmol/L KCl solution and then placed in the reactant solution for the time interval from 0 s to 60 s to test 1-min F deposition. After deposition, the discs were rinsed twice in 50 mL of a stirred solution saturated with respect to CaF_2_ for 5 s. The deposit from each filter disc was extracted by immersing in 1 mL of 0.5 mol/L HClO_4_ solution for 30 min. This solution was then neutralized with 1 mL of Total Ionic Strength Adjuster Background (TISAB II, Orion Research) solution that also contained 0.5 mol/L NaOH, and F concentration was measured by F-electrode (Orion, Mo 94-09). The amount of extracted F from a filter disc was expressed as mass of F per unit surface area of a disc (in µg/cm^2^). The amount of extracted Ca in some selected samples was determined spectrophotometrically as an arsenazo complex using a microanalytical procedure [27]. Particle sizes and morphology of deposited precipitate were examined by JEOL scanning electron microscope (JEOL JSM-5300, JEOL USA, Peabody, MA, USA). Before SEM examination, the filter discs were rinsed for 5 s in solution saturated with respect to and then for 5 s in ethanol.

The 1-min F depositions were determined in 12 mmol/L NaF solutions to which 0.1 mol/L calcium chloride solution was continuously added at average rates of (5, 7.5, 10, 12.5, 15 or 20) mmol L^−1^ min^−1^. The 1-min F deposition was also determined in three different control systems: (I) a two-component F-release rinse that after mixing of equal volumes (75 mL) of components contained 2 mmol/L Na_2_SiF_6_ (12 mmol/L F) and 10 mmol/L CaCl_2_ and 50 mmol/L of acetate buffer at pH = 5, (II) a two-component rinse that after mixing of equal volumes (75 mL) of components contained 12 mmol/L NaF, 10 mmol/L CaCl_2_, and 30 mmol/L KCl, and (III) a conventional one-component 12 mmol/L NaF rinse containing 30 mmol/L KCl.

The data for F depositions in filter discs (*n* = 10) were expressed as mean value ± standard deviation (SD) for each Ca-addition system and control systems. The differences in the mean values (F depositions) among the groups (Ca-release systems and control systems) were tested with one way ANOVA followed by an all pair wise multiple comparison procedure (Student Newman-Keuls method) using the SigmaStat software^©^.

## 3. Results

### 3.1 CaF_2_ Precipitation

**Fig. 2:**
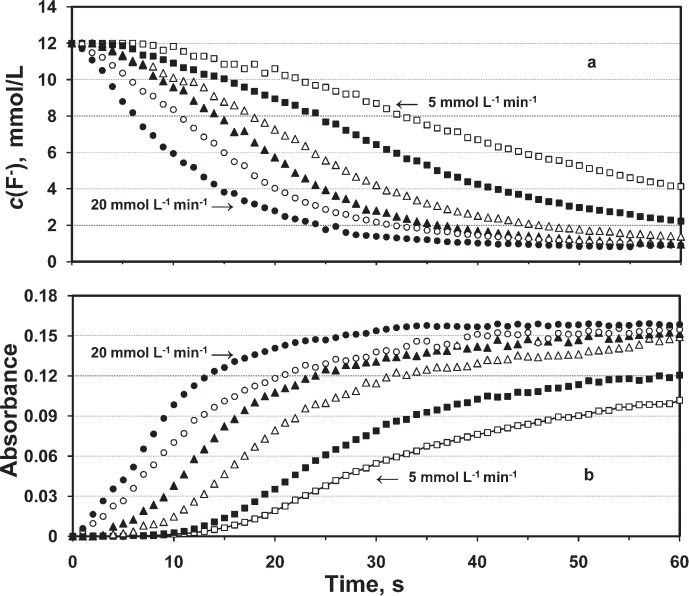
(a) Concentration of fluoride, *c*(F^–^), and (b) absorbance as a function of time for precipitation systems with initial *c*(NaF) = 12 mmol/L, *c*(KCl) = 30 mmol/L, and different Ca addition rates (expressed in mmol L^–1^ min^–1^) of 5(□), 7.5(■), 10(Δ), 12.5(▲), 15(○), and 20 (•).

The changes of *c*(F^−^) in reactant solutions during 1-min Ca addition at the rates of (5 to 20) mmol L^−1^ min^−1^ (*n* = 2 for each system) are shown in Fig. 2a. In all systems *c*(F^−^) continuously decreased with time indicating continuous CaF_2_ precipitation.

The *c*(F^−^) decreases correlated to Ca-addition rates. The increases in *c*(Ca^2+^) in all systems (not shown) were very low at the beginning of reaction whereas in the later times, the increases became proportional to the rates of Ca addition. The pHs of suspensions were 5.8 ± 0.2 and they did not change with time.

Turbidities were very low at the very beginning of the reactions (induction times) indicating the presence of no or very small amounts of CaF_2_ crystals (Fig. 2b). Induction times were from 0 s to 13 s for the systems with Ca-addition rates from (20 to 5) mmol L^−1^ min^−1^, indicating an inverse correlation. After the initial induction period the turbidities continuously increased with time in each of the systems in correlation with increasing Ca-addition rates.

The amounts of the precipitated CaF_2_ calculated from measured *c*(F^−^) in experimental Ca-addition systems showed positive correlation (0.99) with corresponding absorbance values indicating the reliability of these short-term kinetics data.

**Fig. 3:**
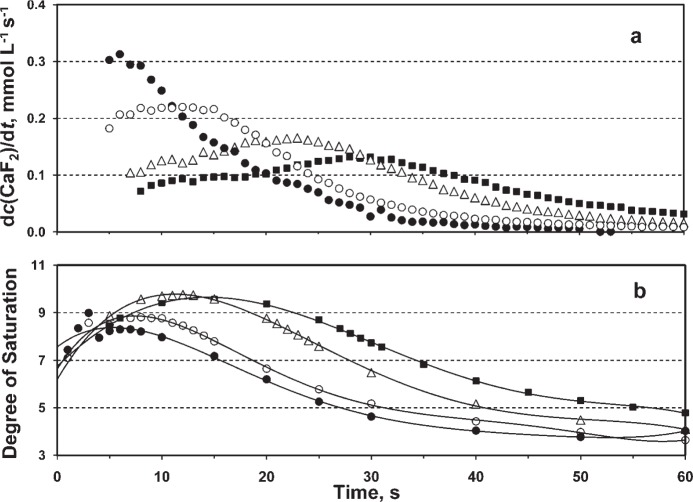
(a) The rate of CaF_2_ precipitation, d*c*(CaF_2_)/d*t* and (b) degree of supersaturation, as functions of time in solutions with *c*(NaF) = 12 mmol/L, *c*(KCl) = 30 mmol/L, and different Ca-addition rates (expressed in mmol L^–1^ min^–1^) of 7.5 (■), 10 (Δ), 15 (○), and 20 (•).

From the amounts of precipitated CaF_2_, the precipitation rates (*R*) expressed as *R* = d*c*(CaF_2_)/d*t* were calculated for all Ca-addition systems. The *R* vs. time curves for selected systems are shown in Fig. 3a.

For Ca-addition rates of (5, 7.5, 10, 12.5, 15, and 20) mmol L^−1^ min^−1^ the maximum precipitation rates (*R*_max_) of (0.10 ± 0.01, 0.13 ± 0.01, 0.16 ± 0.02, 0.20 ± 0.03, 0.22 ± 0.01, and 0.31 ± 0.02) mmol L^−1^ s^−1^ were reached at the times of (33 ± 2, 29 ± 2, 23 ± 2, 18 ± 2, 11 ± 2, and 6 ± 1) s. The *R*_max_ showed positive correlation (0.99) with corresponding Ca-addition rates and negative correlation (0.95) with corresponding times.

The calculated degrees of saturation (*S*) are shown in Fig. 3b. For Ca-addition rates of (5, 7.5, 10, 12.5, 15, and 20) mmol L^−1^ min^−1^, the maximum *S* were 9.2 ± 0.3, 9.6 ± 0.2, 9.8 ± 0.4, 10.3 ± 0.3, 8.8 ± 0.2, and 8.3 ± 0.3, respectively. All *R* vs. time and *S* vs. time curves have similar general trends increasing up to a maximum value and then continuously decreased. In each system the maximum *S* was reached at earlier time than the corresponding maximum *R*.

### 3.2 CaF_2_ Deposition

**Fig. 4:**
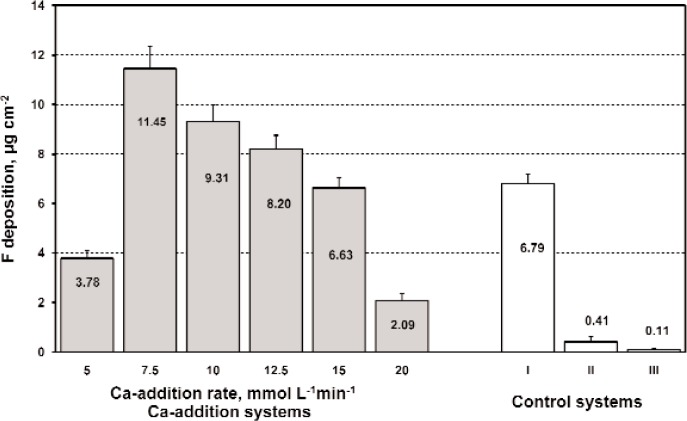
1-min F depositions (mean ± SD, *n* = 10) for Ca-addition system that contained 12 mmol/L NaF solutions with different Ca-addition rates from (5 to 20) mmol L^–1^ min^–1^ and for three control systems: (I) a two-component F release rinse that contained 2 mmol/L Na_2_SiF_6_ (12 mmol/L F), 10 mmol/L CaCl_2_, and 50 mmol/L of acetate buffer at pH = 5, (II) a two component rinse that contained 12 mmol/L NaF, 10 mmol/L CaCl_2_, and 30 mmol/L KCl, and (III) a conventional one component 12 mmol/L NaF rinse containing 30 mmol/L KCl.

For the Ca-addition rates of (5, 7.5, 10, 12.5, 15, and 20) mmol L^−1^ min^−1^, the corresponding 1-min F depositions (mean ± SD, *n* = 10) were (3.78 ± 0.31) µg/cm^2^, (11.45 ± 0.89) µg/cm^2^, (9.31 ± 0.68) µg/cm^2^, (8.20 ± 0.56) µg/cm^2^, (6.63 ± 0.43) µg/cm^2^, and (2.09 ± 0.28) µg/cm^2^, respectively (Fig. 4).

**Fig. 5:**
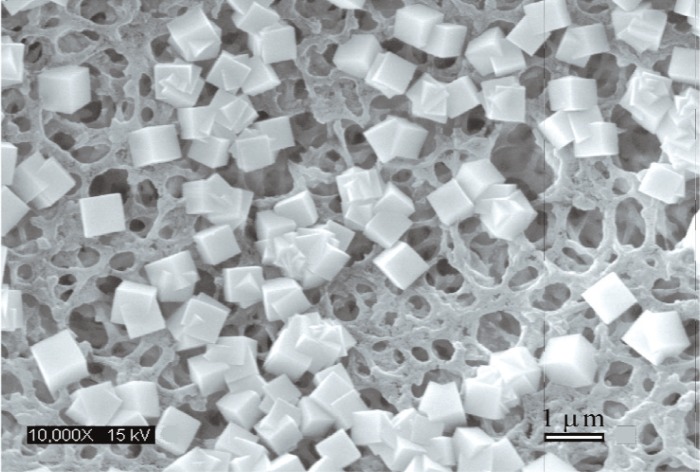
Scanning electron micrographs of CaF_2_ crystals deposited in/on model porous substrate (0.2 µm filter paper) from the solution with *c*(NaF) = 12 mmol/L, *c*(KCl) = 30 mmol/L, and Ca-addition rate of 7.5 mmol L^–1^ min^–1^ at the reaction time of 1 min.

The differences in the F deposition mean values among the systems with different Ca-addition rates were statistically significant (ANOVA, p < 0.05). The Ca/F molar ratio of 0.508 ± 0.013 (*n* = 12, two 1-min deposits from each of six systems were analyzed) indicated precipitation of pure CaF_2_ in all systems. The SEM of the cubical CaF_2_ crystals deposited in/on the model substrate for the system with a Ca-addition rate of 7.5 mmol L^−1^ min^−1^ is shown in Fig. 5.

The 1-min F depositions determined for three control systems (I, II, and III) with the same initial F concentrations (12 mmol/L F) were used for comparison with F deposition from Ca-addition systems. F depositions (mean ± SD, *n* = 10) from control systems (I) two-component F-release rinse (2 mmol/L Na_2_SiF_6_ and 10 mmol/L CaCl_2_), (II) two-component rinse with immediate mixing of F and Ca components (12 mmol/L NaF and 10 mmol/L CaCl_2_), and (III) conventional one-component F rinse (12 mmol/L NaF), were (6.79 ± 0.40) µg/cm^2^, (0.40 ± 0.21) µg/cm^2^, and (0.11 ± 0.02) µg/cm^2^, respectively (Fig. 4). The F deposition mean values for experimental systems with a Ca-addition rate of (7.5, 10, and 12.5) mmol L^−1^ min^−1^ were significantly higher (ANOVA, p < 0.05) than F deposition mean values for control systems I, II, and III.

## 4. Discussion

The data on *c*(F^−^) changes with time in the reactant solutions indicated that the rates of CaF_2_ precipitation (*R*) are controlled by the rates of Ca addition. This result is supported by the turbidity data, where an increase in turbidity correlated to the formation of CaF_2_ particles.

All *R* vs. time curves have similar general trends increasing up to a maximum rate of precipitation value and then continuously decreased with time (Fig. 3a). All calculated log*R* vs. time and log*R* vs. −log*S* curves (not shown) have similar trends as the *R* vs. time curves showing the continuous increase initially, and the continous decrease in the later times. The increase of log*R* with −log*S* curve up to the maximum value is typical for the time interval in which nucleation and crystal growth are concomitant processes and the further linear decrease of log*R* with −log*S* indicates that crystal growth was dominant in that time interval [28].

These precipitation kinetics data could be used for the explanation of the F deposition data. For the experimental systems with moderate Ca-addition rates of (7.5, 10, and 12.5) mmol L^−1^ min^−1^ that produced significantly larger F depositions, the maximum precipitation rates (*R*_max_) of (0.13, 0.17 and 0.20) mmol L^−1^ s^−1^ were reached in (29, 23, and 18) s, respectively. It indicated that the continuous nucleation and relatively slow crystal growth during the time intervals (0 to 29)s, (0 to 23) s, and (0 to 18) s for Ca-addition rates of (7.5, 10, and 12.5) mmol L^−1^ min^−1^, respectively, produced crystals that were small enough to penetrate in the small substrate pores. The majority of these single CaF_2_ crystals that were initially formed or penetrated in/on the model substrate could be later fixed in surface pores by inter-growth and/or agglomeration (Fig. 5). For the experimental system with the highest Ca-addition rate of 20 mmol L^−1^ min^−1^ that produced very low F deposition, *R*_max_ of 0.31 mmol L^−1^ s^−1^ was reached in 6s. It indicated that CaF_2_ crystal growth almost immediately became dominant process in this system (Fig. 3a, decrease of *R* vs. time curve after 6s) and correspondingly, the CaF_2_ crystals became too large for deposition in/on the porous model substrate. It is important to note that even lower F deposition was determined from the control system II with immediately mixed Ca and F components. It could be explained by an extremely fast growth and agglomeration of formed crystals that instantaneously became too large to penetrate in/on much smaller substrate pores. The still lower F deposition from the one-component conventional F rinse (control system III) could be explained by the absence of calcium in reactant solution and, therefore, the adsorption of F ions was the only possible F deposition mechanism.

The cubical shape of CaF_2_ crystals (Fig. 5) formed in these Ca-addition systems is typical for precipitation from pure fluoride solutions. The CaF_2_ crystals formed in fluorosilicate solutions (control system I) had spherical shape, similar as crystals formed in phosphate solutions [29,30].

The 1-min CaF_2_ depositions in experimental systems do not positively correlate to the CaF_2_ precipitation rates. The largest CaF_2_ depositions were obtained from the Ca-addition systems in which precipitation rates moderately increased with time and reached maximum values after prolonged time intervals. It seems that continuous formation and penetration of submicron-size crystals during these time intervals were prerequisites for large 1-min CaF_2_ depositions. These results indicate that in the F systems with continuous Ca addition, the rate of CaF_2_ precipitation that controls CaF_2_ deposition is critical factor for deposition effectiveness.

## 5. Conclusions

The rate of Ca addition controls rate of CaF_2_ precipitation and deposition. The CaF_2_ precipitation rates are proportional to the Ca-addition rates but the CaF_2_ depositions are not proportional to the Ca-addition rates. The CaF_2_ depositions from 12 mmol/L NaF solutions with continuous Ca-addition rates of 7.5 mmol L^−1^ min^−1^ to 12.5 mmol L^−1^ min^−1^ having maximum precipitation rates of 0.13 mmol L^−1^ s^−1^ to 0.20 mmol L^−1^ s^−1^, respectively, are significantly higher than depositions in systems without continuous Ca addition.

## Footnotes

1 Certain commercial materials and equipment are identified in this paper to specify the experimental procedure. In no instance does such identification imply recommendation or endorsement by the National Institute of Standards and Technology, the National Institutes of Health, or the ADA Foundation or that the material or equipment identified is necessarily the best available for the purpose.
